# Assessment and prediction of road accident injuries trend using time-series models in Kurdistan

**DOI:** 10.1186/s41038-018-0111-6

**Published:** 2018-03-09

**Authors:** Maryam Parvareh, Asrin Karimi, Satar Rezaei, Abraha Woldemichael, Sairan Nili, Bijan Nouri, Nader Esmail Nasab

**Affiliations:** 1 Social Determinants of Health Research Center, Kurdistan University of Medical Sciences, Sanandaj, Iran; 2 Research Center for Environmental Determinants of Health, Kermanshah University of Medical Sciences, Kermanshah, Iran; 3 School of Public Health, College of Health Sciences, Mekelle University, Tigray, Ethiopia; 4 Department of Epidemiology and Biostatistics, School of Public Health, Kerman University of Medical Health, Kerman, Iran

**Keywords:** Road accidents, Prediction, Time-series models

## Abstract

**Background:**

Road traffic accidents are commonly encountered incidents that can cause high-intensity injuries to the victims and have direct impacts on the members of the society. Iran has one of the highest incident rates of road traffic accidents. The objective of this study was to model the patterns of road traffic accidents leading to injury in Kurdistan province, Iran.

**Methods:**

A time-series analysis was conducted to characterize and predict the frequency of road traffic accidents that lead to injury in Kurdistan province. The injuries were categorized into three separate groups which were related to the car occupants, motorcyclists and pedestrian road traffic accident injuries. The Box-Jenkins time-series analysis was used to model the injury observations applying autoregressive integrated moving average (ARIMA) and seasonal autoregressive integrated moving average (SARIMA) from March 2009 to February 2015 and to predict the accidents up to 24 months later (February 2017). The analysis was carried out using R-3.4.2 statistical software package.

**Results:**

A total of 5199 pedestrians, 9015 motorcyclists, and 28,906 car occupants’ accidents were observed. The mean (SD) number of car occupant, motorcyclist and pedestrian accident injuries observed were 401.01 (SD 32.78), 123.70 (SD 30.18) and 71.19 (SD 17.92) per year, respectively. The best models for the pattern of car occupant, motorcyclist, and pedestrian injuries were the ARIMA (1, 0, 0), SARIMA (1, 0, 2) (1, 0, 0)_12_, and SARIMA (1, 1, 1) (0, 0, 1)_12_, respectively. The motorcyclist and pedestrian injuries showed a seasonal pattern and the peak was during summer (August). The minimum frequency for the motorcyclist and pedestrian injuries were observed during the late autumn and early winter (December and January).

**Conclusion:**

Our findings revealed that the observed motorcyclist and pedestrian injuries had a seasonal pattern that was explained by air temperature changes overtime. These findings call the need for close monitoring of the accidents during the high-risk periods in order to control and decrease the rate of the injuries.

## Background

Road traffic accidents are associated with high mortality, severe injuries, and considerably high economic losses globally [1]. The road traffic accidents account for 12% of the burden of disease and to the high rates of unintentional injuries [2]. About 1.2 million deaths and more than 50 million injuries occur annually. Therefore, road traffic accident is one of the most common causes of mortality and disability in the world [3, 4]. Evidences from Iran on the disability-adjusted life year (DALY) index indicated that road traffic accident was ranked ninth in 1990, and is expected to be the third by 2020 [5].

The pedestrians, cyclists, and motorcyclists were particularly at high-risk of road traffic accidents and more than 90% of the deaths from the accidents occurred in low-income countries [6]. The death rate in the developing countries has been on the rise while it was continuously decreasing in the developed countries. Currently, the car ownership in most Asian countries has increased twice faster than the per capita income [7], and this might have contributed to the increasing rates of accidents in the region.

The death rate of road traffic accidents in Iran in 2002 which was at 44 per 100,000 populations was more than twice the global average (19 per 100,000) [8]. The annual DALY loss from road traffic accidents was more than 1,200,000 per year [4]. This event accounted for 57% of the DALY lost and more than 80% of the victims were among males of 15–29 years [9].

Since the past seven decades, the deaths from road traffic accidents in Iran have been increasing. The mortality trend during the years 2001–2005 reached as high as 28,000 deaths per year [10]. Despite the reduction in mortality from 38 per 100,000 in 2006 to 31 per 100,000 in 2008, the number of injured people from road traffic accident has been increasing overtime [11, 12]. Thus, the mortality of road traffic accidents in Iran remained among the highest rates in the world [13]. Despite all interventions to reduce traffic accident injuries, even fatal injuries have still a high incidence rate in Iran [12].

The geographic, environmental, demographic, and individual factors can contribute to the occurrence of road traffic accidents. Hence, access to the information about road traffic accidents in a given context is significant to generate evidence to contribute to the prevention and control of context-specific accidents [3]. The objective of this study was modeling the frequency of injuries due to road accidents in Kurdistan Province, located in the west of Iran, from March 2009 to February 2015 and to predict injuries until February 2017 using time series models.

## Methods

### Study design and population

This cross-sectional study was conducted to analyze time-series observations of traffic accident injuries from March 2009 to February 2015 in Kurdistan Province. The data was obtained from the Accidents’ Unit of the Health Deputy in the Kurdistan University of Medical Sciences. The unit regularly gathers data on road traffic accident injuries from hospitals, police stations, forensic medicine, and road organization. In order to ensure the quality of the collected data, any duplicate or redundant information concerning the injuries was cleaned. Then, the data on the injured persons was divided into three separate categories: pedestrian injuries, motorcyclist injuries, and car occupant injuries and each category were analyzed separately.

### Data analysis

The time-series analysis was applied to model the observed frequency of injuries in the study area and to predict the future incidence. The Box-Jenkins approach was used to develop an autoregressive integrated moving average (ARIMA) model. The monthly time-series observation was used to increase the prediction power of the model. The ARIMA model was expressed by ARIMA (p, d, q), where the p, d, and q represented the number of ordinary autoregressive, differences, and moving average parameters, respectively. In other words, the p and q were the number of significant lags of the autocorrelation function (ACF) and the partial autocorrelation function (PACF) plots, respectively, and d was the different order needed to remove the ordinary non-stationarity in the mean of the error terms. Furthermore, a seasonal autoregressive integrated moving average (SARIMA) model notation of SARIMA (p, d, q) (P, D, Q) _S_, where S referred to the seasonal periods, P and Q to the number of SARIMA parameters (number of consecutive seasons that were correlated), respectively, and D to the different order needed to remove the seasonal non-stationary series in the model. The equations of these two models are as follows:

$$ \mathrm{ARIMA}\ \left(p,d,q\right):\kern1.25em {\varphi}_p\left(\beta \right){\left(1-\beta \right)}^d{Z}_t={\theta}_q\left(\beta \right){a}_t $$

$$ \mathrm{SARIMA}\ \left(p,d,q\right){\left(P,D,Q\right)}_s:\kern1.25em {\Phi}_P\left({\beta}^s\right){\varphi}_p\left(\beta \right){\left(1-{\beta}^s\right)}^D{\left(1-\beta \right)}^d{Z}_t={\Theta}_Q\left({\beta}^s\right){\theta}_q\left(\beta \right){a}_t $$

Where *φ*_*p*_(*β*) = (1 − *φ*_1_*β* − … − *φ*_*p*_*β*^*p*^), *θ*_*q*_(*β*) = (1 − *θ*_1_*β* − … − *θ*_*q*_*β*^*q*^), Φ_*P*_(*β*^*S*^) = (1 − Φ_1_*β*^*S*^ − … − Φ_*P*_*β*^*Ps*^) and Θ_*Q*_(*β*^*s*^) = (1 − Θ_1_*β*^*S*^ − … − Θ_*Q*_*β*^*Qs*^). In these extensions, *φ* and *θ* are the ordinary autoregressive and moving average parameters; and, Φ and Θ are the seasonal autoregressive and moving average parameters. Besides, *β* represents the time-delay parameter so that *β*^*r*^*Z*_*t*_ is equal to *Z*_*t* − *r*_.

The Box-Jenkins modeling involved several steps, including model identification, parameter estimation, checking, and prediction [14]. The first step included testing for “white noise” in the data using the Portmanteau and Bartlett test. The stationarity in the seasonal and non-seasonal series was checked by Dickey-Fuller test. For the SARIMA model construction, the order of (p, d, q) (P, D, Q) was distinguished based on the ACF and PACF plots. The cutoff point for the ACF plot and the slow decay in the PACF plot were used to identify the correct parameters. The model parameters were estimated using ordinary least-squares approach and *t*-test was considered to decide on the significant difference [15]. Based on the Box-Jenkins approach, the most parsimonious SARIMA model that requires the least moving average and autoregressive parameters should be selected.

The Akaike Information Criteria (AIC) and Schwartz Bayesian Criteria (SBC) were calculated to evaluate the goodness of fit for each model [16]. These indicators evaluated the model fitness based on the likelihood model and a number of parameters. The smaller the size, the better was the model. The absence of process autocorrelation and partial autocorrelation in the residuals are also indicators of the model’s goodness of fit. Finally, the fitted model was used to predict the trend of road accident injuries from February 2015 to February 2017 in the context. All the analyses and the forecast were computed using the R statistical software package. The statistical significance was decided at *p* < 0.05.

## Results

Over the 72 months observation (from March 2009 to February 2015), a total of 43,120 road traffic accident injuries were reported and 5199 (3426 male, 1773 female) of which were pedestrians, 9015 were motorcyclists, and 28,906 were car accidents, respectively (Table 1). The number of injured pedestrian per month varied from 20 to 186 with a mean 72.20 and standard deviation (SD) of 33.77. The number of injured motorcyclists ranged from 15 to 268 per month and the mean was 125.20 (SD 64.84). Similarly, the number of injured car occupants ranged from 211 to 656 per month with a mean of 401.47 (SD 112.94). However, the highest number of pedestrian (*n* = 1252) and motorcyclist (*n* = 2174) injuries were observed during 2014. The mean car occupant injuries observed was 401.47 (SD 112.94). The highest number of car occupant injuries (*n* = 5485) was observed in 2010 while the least (*n* = 4369) was observed in 2011.

**Table 1:** Frequencies of pedestrians’, motorcyclists’, and car occupants’ injuries from March 2009 to February 2015

	Frequency of injuries per year (*n)*							
Type of accident	2009 (Mar–Dec)	2010	2011	2012	2013	2014	2015 (Jan–Feb)	Total
Pedestrians	644	652	701	1087	700	1252	163	5199
Motorcyclists	1249	1327	1466	1550	919	2174	330	9015
Car occupants	3831	5485	4369	4472	5155	5073	521	28,906
Total	5724	7464	6536	7109	6774	8499	1014	43,120

### Seasonality of pedestrian accident injuries

**Fig. 1:**
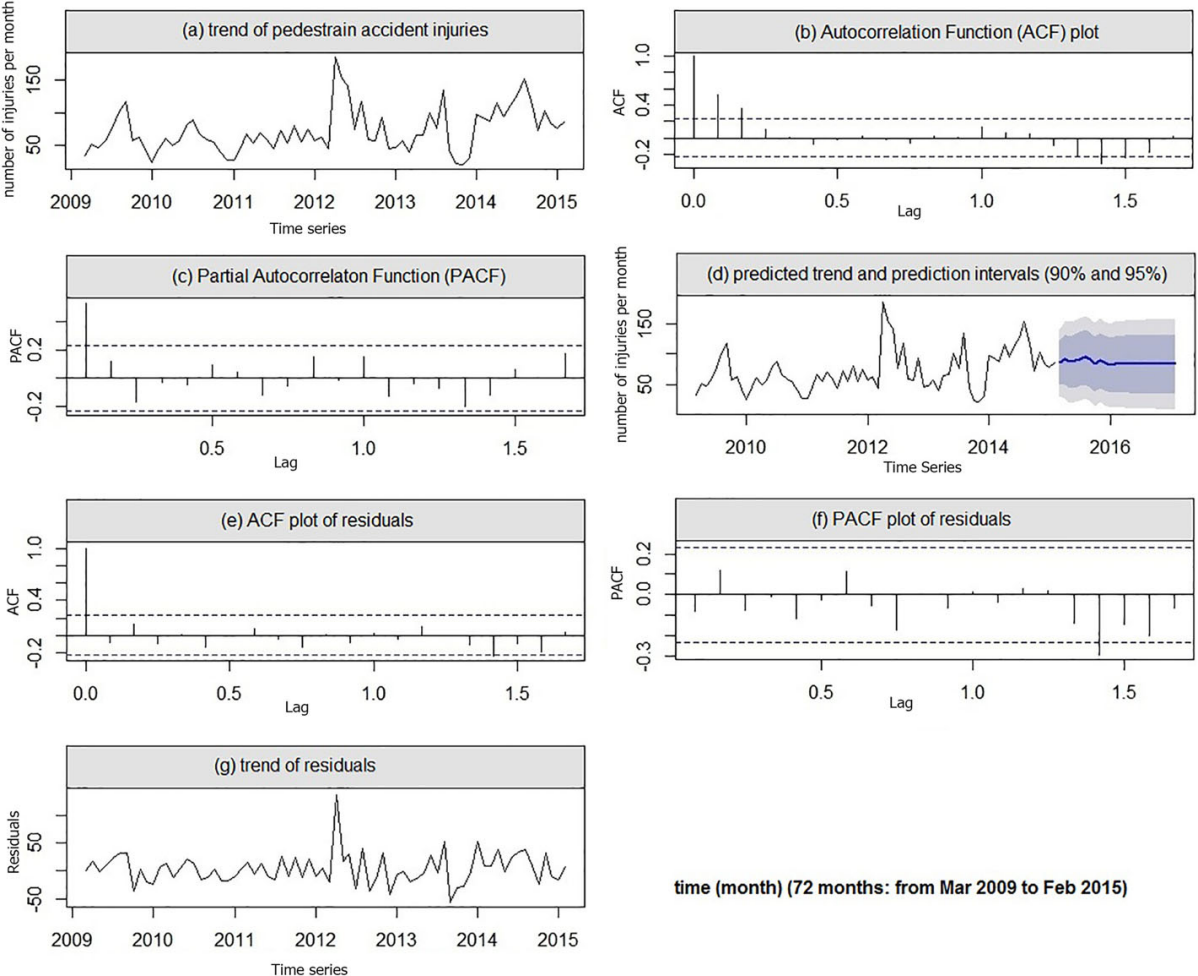
The trend of pedestrian injuries (**a**), and autocorrelation function (ACF) and the partial autocorrelation function (PACF) plots (**b** and **c**) show a seasonal pattern in data. ACF and PACF lags of residuals (**e** and **f**) do not exceed the dash line, and random walk trend of residuals (**g**) indicates the goodness of predicted models (**d**)

Pedestrian accident injuries varied over the study period as shown in Fig. 1a. The frequency of pedestrian injuries displayed seasonality which was repeated annually. The frequency was low during winter, increased during the spring, and peaked in the late summer (August). The lowest injury frequency was observed during January. The annual cycle of the number of pedestrian injuries plotted in Fig. 1a displayed a sine function at a 12-month period. Despite the ACF and PACF shown in Fig. 1b, c reflected a non-stationarity in the mean, which was confirmed by Dickey-Fuller test (*p* = 0.194), the first-order difference of the data showed a stationary pattern. The final model for pedestrian accident injuries was SARIMA (1, 1, 1) (0, 0, 1)_12_ with the following equation which showed the best goodness of fit (Table 2).

1 $$ {Z}_t=1.54{Z}_{t-1}-0.54{Z}_{t-2}+{a}_t-0.96{a}_{t-1}-0.19{a}_{t-12}+0.19{a}_{t-13} $$

**Table 2:** Parameter estimations and goodness of fit measures for pedestrians’, motorcyclists’, and car occupants’ injuries from Mar 2009 to Feb 2015

	Parameters	Coefficient	SE	AIC	BIC
Pedestrians’ injuries	AR1	0.542	0.115	684.98	694.03
	MA1	− 0.962	0.047		
	SMA1	0.198	0.111		
Motorcyclists’ injuries	AR1	0.623	0.143	753.13	766.79
	MA1	− 0.173	0.180		
	MA2	0.371	0.116		
	SAR1	0.411	0.126		
Car occupants’ injuries	AR1	0.604	0.093	857.61	864.44

*AR*_*i*_ and *MA*_*i*_ are the *i*th order of autocorrelation and partial autocorrelation, respectively. Also, *SAR*_*i*_, and *SMA*_*i*_ are the *i*th order of seasonal autocorrelation and seasonal partial autocorrelation, respectively. *AIC* Akaike Information Criterion *BIC*Bayesian Information Criterion

The Portmanteau test of the residuals for this model could not reject the null hypothesis that the model does not indicate any lack of fit (*p* > 0.05). The ACF, PACF, and the trend for the modeled residuals presented in the Fig. 1e–g suggested that the residuals are white noise. We predicted the number of pedestrian injuries using Eq. (1) until February 2017 (Fig. 1d and Table 3).

**Table 3:** Predicted frequencies of pedestrians’, motorcyclists’, and car occupants’ injuries from March 2015 to February 2017

Date	Pedestrians (*n*)	Motorcyclists (*n*)	Car occupants (*n*)
Mar 2015	85.31	161.70	322.06
Apr 2015	90.85	174.36	351.73
May 2015	86.28	155.15	369.68
Jun 2015	88.42	159.11	380.53
Jul 2015	91.83	190.21	387.10
Aug 2015	94.97	174.96	391.07
Sep 2015	91.17	168.57	393.47
Oct 2015	82.81	139.09	394.92
Nov 2015	89.15	139.92	395.80
Dec 2015	84.55	141.73	396.33
Jan 2016	80.80	139.51	396.65
Feb 2016	82.86	148.48	396.85
Mar 2016	82.81	142.53	396.97
Apr 2016	82.79	147.70	397.04
May 2016	82.77	139.77	397.08
Jun 2016	82.77	141.38	397.11
Jul 2016	82.76	154.18	397.12
Aug 2016	82.76	147.90	397.13
Sep 2016	82.76	145.26	397.14
Oct 2016	82.76	133.12	397.14
Nov 2016	82.76	133.46	397.14
Dec 2016	82.76	134.20	397.14
Jan 2017	82.76	133.29	397.14
Feb 2017	82.76	136.98	397.15

### Seasonality of motorcyclist’s accident injuries

**Fig. 2:**
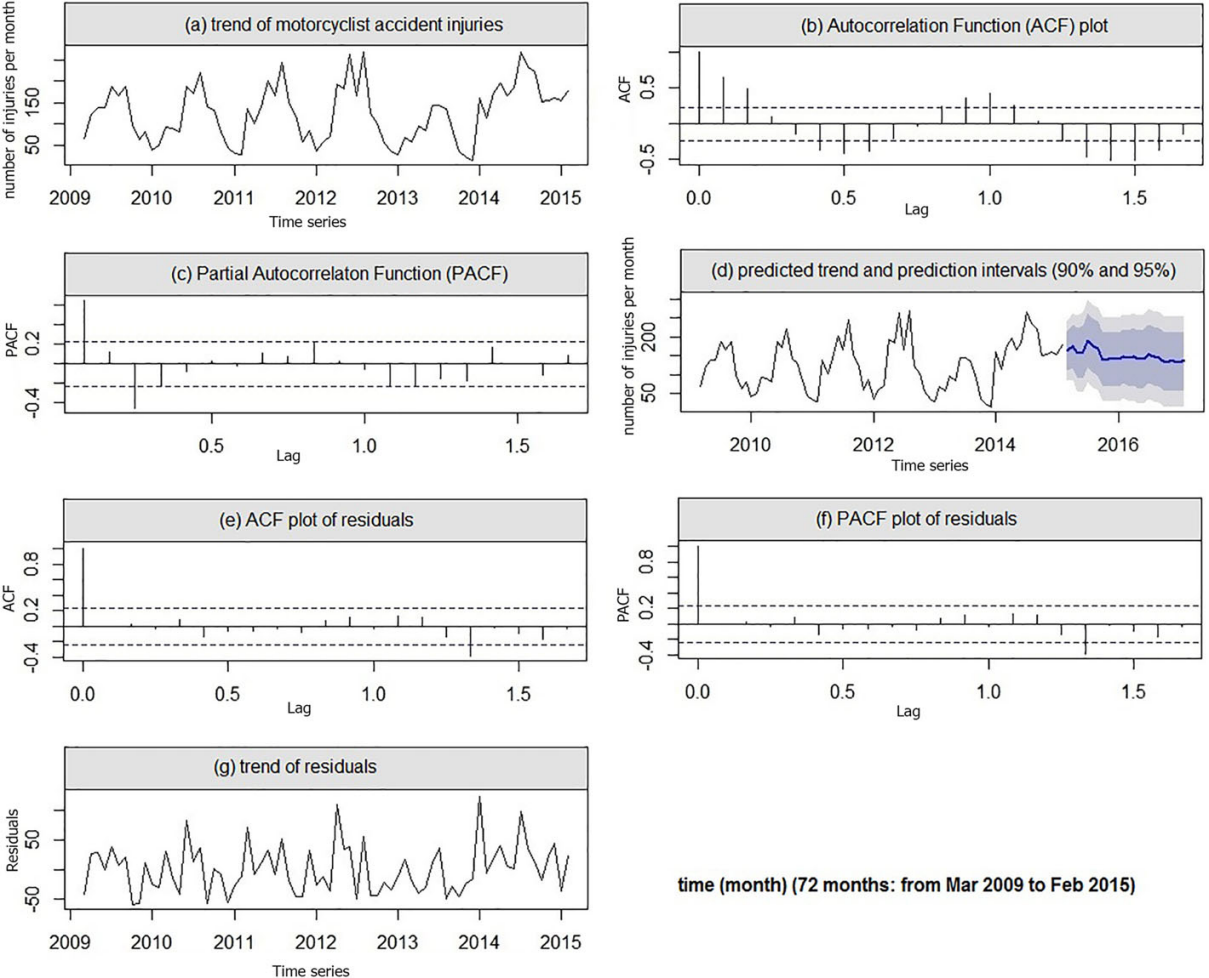
The trend of motorcyclist injuries (**a**), and autocorrelation function (ACF) and the partial autocorrelation function (PACF) plots (**b** and **c**) show a seasonal pattern in data. ACF and PACF lags of residuals (**e** and **f**) do not exceed the dash line, and random walk trend of residuals (**g**) indicates the goodness of predicted models (**d**)

The time-series observations of the frequency of the motorcyclist accident injuries had a positive autocorrelation phenomenon among the consecutive months which was depicted in Fig. 2a. Similar to the model of the pedestrian injuries, the frequency of the motorcycle injuries displayed a seasonality that was repeated annually. The frequency of the motorcyclists’ injuries was low in the winter, rose during the spring and peaked during the late summer (August). The minimum frequency of the accidents was observed during December. Furthermore, Fig. 2a depicted the year to year seasonality pattern in the frequency of the injuries which could be apparently recognized from the ACF and the PACF plots (Fig. 2b, c). The results of the Dickey-Fuller test (*p*= 0.030) with pattern (Fig. 2a–c) indicated stationarity of the residuals of the motorcyclists’ accident injury time-series observations. Therefore, after comparing the different orders of the AR and the MA parameters, SARIMA (1, 0, 2) (1, 0, 1)_12_, the model was fitted to predict the frequency of the motorcyclists’ accident injuries (Table 2). The model can be written mathematically as the follows:

2 $$ {Z}_t=0.62{Z}_{t-1}+0.41{Z}_{t-12}-0.25{Z}_{t-13}+{a}_t+0.17{a}_{t-1}-0.37{a}_{t-2} $$

The residuals’ ACF, PACF, and trend plots exhibited a white noise pattern and the goodness of the fitted model (Fig. 2e–g). Finally, the frequency of motorcyclists’ accident injuries was predicted until February 2017 using the above mentioned Eq. (2) (Fig. 2d, Table 3).

### Car occupants’ injuries in car accident

**Fig. 3:**
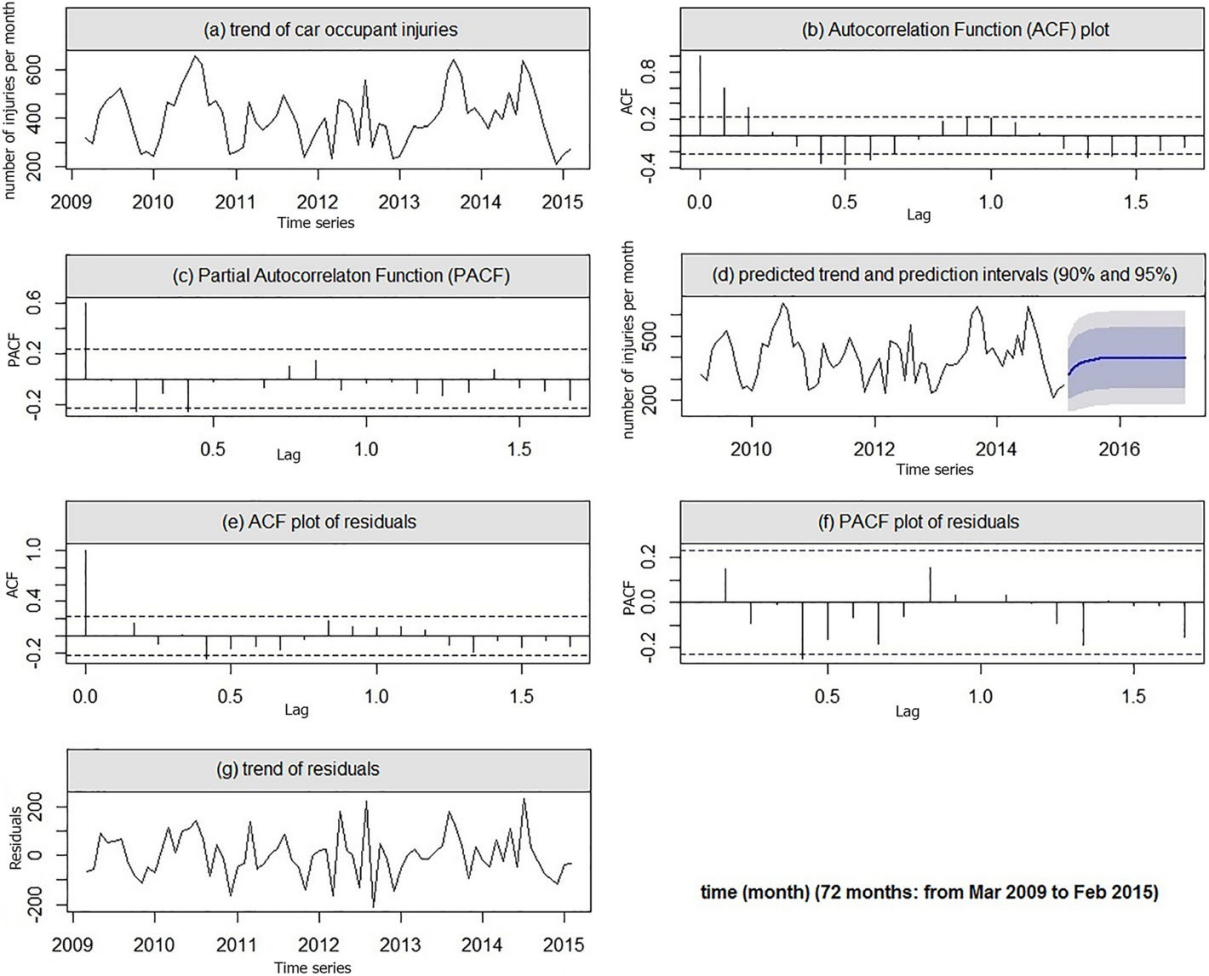
The trend of car occupant injuries (**a**), and autocorrelation function (ACF) and the partial autocorrelation function (PACF) plots (**b** and **c**) show a seasonal pattern in data. ACF and PACF lags of residuals (**e** and **f**) do not exceed the dash line, and random walk trend of residuals (**g**) indicates the goodness of predicted models (**d**)

The trends of the total car occupant injuries (*n* = 28,906) observed during the period 2009 to 2015 is shown in Fig. 3a. However, the frequency of the injuries was a non-seasonal time-series data and the frequency was low during the winter, rose during the spring, and peaked in the late summer (August). The frequency of the injuries was minimum during the December. The Dickey-Fuller test showed stationarity of the times-series observations (*p*= 0.006). Finally, the selected model for the car occupant injuries using the ACF and PACF plots (Fig. 3b, c) was ARIMA (1, 0, 0), and the equation is presented as follows (Table 2):

3 $$ {Z}_t=0.604{Z}_{t-1}+{a}_t $$

The model’s goodness of fit was confirmed based on the residuals’ behavior as shown in Fig. 3e–g, showed the predicted frequency of the car occupants’ injuries for the 24 months period using the model which was presented in Eq. 3 is shown in Table 3 and Fig. 3d.

## Discussion

This study applied the time-series modeling to determine and predict road traffic accident injuries patterns in Kurdistan Province, Iran. Time-series analysis was a widely used method for modeling phenomena [17]. Several studies also applied this model to predict the road traffic accident fatal injuries [12, 18, 19] and were proven effective for determining accident prevention strategies in a short time [12]. The remarkably high frequency of road traffic injuries (*n* = 43, 120 cases) observed in our study in about 6 years indicated the public health importance of the issue. Despite the report of a descending trend of deaths from road traffic accident [20], our findings indicated an increasing trend in the frequency of non-fatal injuries. Another study in Kermanshah Province also indicated a seasonally descending trend of deaths between the years 2006 and 2013 [21]. More than 67% occupant injuries and about 12% pedestrian injuries were the maximum and minimum frequency of road traffic injuries. The predicted frequency of car occupant injuries during the period 2015 to 2016 revealed a high rate of injuries. These findings were not consistent with the findings of another study during the years 2008 to 2014 which reported a decrease trend of the car occupants’ injuries [22]. The reductions in the deaths from accidents can be the consequence of extensive and coordinated efforts of several organizations that bold the importance of some interventions and strict monitoring the implementation of the interventions. The interventions included the mandatory use of seat belt by drivers and passenger vehicles, helmet use by motorcyclists, improving the quality of vehicles and roads, and public education programs aimed at prevention of accidents [23, 24]. These interventions can reduce the occurrence of death from the road traffic accidents, but may not completely prevent injuries.

Despite the reducing trend in the death rate from road traffic accidents, evidence showed that Iran was one of the countries with high incidence of road traffic accidents [24]. There were 23,249 fatal road traffic injuries in 2013 in Iran [25] which was more than seven times the report from Germany (3648) and about six times more than that in Turkey (4045) [25]. The extremely high incidence of road accidents in Iran may relate to geographic factors, low cost of gasoline, poor public transportation system, rapidly growing automotive industry, poor quality of cars and motorcycle equipment, and unsafe roadway networks [8].

The trend of motorcycle injuries in this study was extremely increasing overtime. The high price of cars and the heavy traffic jams, might have contributed to the increased number of individuals who used motorcycle for transportation. Thus, the increasing trend of injuries might be associated with an increased number of motorcycle riders. Epidemiologic studies indicated that most accidents due to motorcyclists were related to younger men and the low-income groups [26, 27]. However, there is concrete evidence concerning the factors related to motorcyclist accidents in Iran [27]. Hospital-based evidence from USA indicated an increasing number of motorcyclist accident during the years 2001 to 2008 regardless of their age [28]. Others reported forward inattention, yield non-conformity, and moving in the opposite direction as the most common causes of motorcyclist accidents [29]. The helmet use was one of the effective ways of decreasing the likelihood of death and head injuries among motorcyclist [30]. According to the 2010 WHO report, only 30% motorcycle riders and 10% of motorcycle passengers use a helmet [27]. In a study conducted in Kerman Province, it was also found that from total of 2880 motorcyclists, only 324 (11.2%) used a helmet and 5% of them were passengers [31].

As you can see in Figs. 1, 2, and 3, our predicted trends in the number of injuries after February 2015 is so smooth, this case may be due to small autocorrelation and partial autocorrelation coefficients of the fitted models (Table 2). Because small-value coefficients make the process of injuries similar to a random walk model which it returns a flat forecast function.

Our study followed 12-month seasonal pattern of injuries and most of the car occupants’ injuries were predicted to occur in August and September. The evidences from other studies also showed similar findings [32, 33]. A study in Esfahan Province found that most of the motorcycle accidents occurred during the August and the least occurred during the December [34], while a report from a meta-analysis indicated that the majority of the motorcycle accidents in Iran occurred during the first half of the year in the evening [27]. The large number of motorcycle injuries during August in our study might be related to the low helmet use during the hot season. Furthermore, it might be related to the high mobility of people during summer season usually for holidays [10].

Kurdistan Province is geographically mountainous, and the road networks connect it with other provinces in the northern, western, and southern parts of Iran. A highly busy roadway can also increase the risk of human factors that lead to road traffic accidents. The road traffic accidents in a mountainous province can be highly fatal [11]. Thus, the road traffic accident prevention intervention programs and periodic evaluation of those actions can help reduce the incidence of accidents.

## Conclusion

Our findings indicated increasing trends of the car occupants’ and motorcyclists’ injuries in Kurdistan Province during the period of 2009 to 2017. The majority of the road traffic injuries occurred during the summer season. The decision-makers and planners in Iran can draw advantage of the findings for planning the prevention and control measures of road traffic injuries in the context.

## References

[1] Hesari A, Esmaeli A (2004). Estimates of deaths from traffic accidents on life expectancy at birth and the financial burden it (2002). Health Inf Manag.

[2] Marasy MR, Tabar IM (2013). The burden of road traffic injuries in Isfahan, Iran in 2010. J Kerman Univ Med Sci.

[3] Yazdani CJ, Ahmadi BE, Ghadami M (2012). Mapping of mortality rate in suburban accidents, Mazandaran Province, 2007-2010. J Mazandaran Univ Med Sci.

[4] Ghorbani A, Nabavi fard H, Khoshhal M, Hosseini H (2011). Costs imposed on the effects of mortality due to traffic accidents (Sabzevar). Traffic Manag Stud.

[5] Ayatollahi SH, Hassanzadeh J, Ramezani A (2009). The burden of traffic accidents in South Khorasan Province, Iran in 2005. Iran J Epidemiol..

[6] Herman J, Ameratunga S, Jackson R (2012). Burden of road traffic injuries and related risk factors in low and middle-income Pacific Island countries and territories: a systematic review of the scientific literature (TRIP 5). BMC Public Health.

[7] Mohan VR, Sarkar R, Abraham VJ, Balraj V, Naumova EN (2015). Differential patterns, trends and hotspots of road traffic injuries on different road networks in Vellore district, southern India. Tropical Med Int Health.

[8] Naghavi M, Shahraz S, Bartels D, Puthenpurakal JA, Motlagh ME (2009). Adverse health outcomes of road traffic injuries in Iran after rapid motorization. Arch Iran med.

[9] Naghavi MA, Abolhassani F, Pourmalek F, Jafari N, Moradi LM, Eshrati B (2008). The burden of disease and injury in Iran 2003. Iran J Epidemiol.

[10] Moradi A, Kh R (2014). Trend of traffic accidents and fatalities in Iran over 20 years (1993-2013). J Mazandaran Univ Med Sci.

[11] Ghadirzadeh MR, Shojaei A, Khademi A, Khodadoost M, Kandi M, Alaeddini F (2015). Status and trend of deaths due to traffic accidents from 2001 to 2010 in Iran. Iran J Epidemiol.

[12] Bakhtiyari M, Mehmandar MR, Riahi SM, Mansournia MA, Sartipi M, Bahadorimonfared A (2016). Epidemiologic pattern of fatal traffic injuries among Iranian drivers; 2004–2010. Iran J Public Health.

[13] Moradi A, Rahmani K, Hoshmandi-Shoja M, Rahimi-Sepehr H, Khorshidi A (2016). An overview of the situation of traffic accidents in Iran in comparison with other countries. Iran J Forensic Med.

[14] Duenas C, Fernandez MC, Canete S, Carretero J, Liger E (2005). Stocastic model to forecast ground level ozone concentration at urban and rural areas. Chemosphere.

[15] Lin Y, Chen M, Chen G, Wu X, Lin T (2015). Application of an autoregressive integrated moving average model for predicting injury mortality in Xiamen. China BMJ Open.

[16] Box GEP, Jenkins GM, Reinsel GC, Ljung JM, David JB, Noel AC, Garrett MF (2016). Time series analysis: forecasting and control.

[17] Mansouri F, Khanjani N, Rananadeh KL, Pourmousa R (2013). Forecasting air pollutant situation using the time series models in Kerman, Iran. Sci J School Pub Health Ins Pub Health Res.

[18] Bahadorimonfared A, Soori H, Mehrabi Y, Rahmati RM, Esmaili AR, Salehi M (2013). Trends of fatal road traffic injuries in Iran (2004–2011). PLoS One.

[19] Bahadori MA, Soori H, Mehrabi Y, Rahmati RM, Esmaili AR, Salehi M (2013). A model for prediction of on the rate of mortality due to road traffic accidents in Iran. Res Med.

[20] Mehmandar MS, Soori H, Mehrabi Y (2016). Predicting and analyzing the trend of traffic accidents deaths in Iran in 2014 and 2015. Int J Crit Illn Inj Sci.

[21] Zolala F, Haghdoost AA, Ahmadijouybari T, Salari A, Bahrampour A, Baneshi MR (2016). Forecasting the trend of traffic accident mortality in West Iran. Health Scope.

[22] Yousefzadeh CS, Ranjbar TF, Malekpouri R, Razzaghi A (2016). A time series model for assessing the trend and forecasting the road traffic accident mortality. Arch Trauma Res.

[23] Rasouli MN, Nouri M, Zarei MR, Saadat S, Rahimi MV (2008). Comparison of road traffic fatalities and injuries in Iran with other countries. Chin J Traumatol.

[24] Soori H, Royanian M, Zali AR, Movahedinejad A (2009). Study of changes on road traffic injury rates, before and after of four interventions by Iran traffic police. Pajoohandeh J.

[25] Soori H, Iranfar M (2013). Road traffic status in the world and Iran: review of results from the World Health Organization. J Saf Prom Inj Prev.

[26] Mirzaei M, Mirzadeh M, Shogaei-Far H, Mirzaei M (2016). Trends in road traffic deaths in Yazd, Iran, 2004 - 2010. Arch Trauma Res.

[27] Sadeghi H, Ayubi E, Azami-Aghdash S, Abedi L, Zemestani A, Amanati L (2016). Epidemiological patterns of road traffic crashes during the last two decades in Iran: a review of the literature from 1996 to 2014. Arch Trauma Res.

[28] Jackson TL, Mello MJ (2013). Injury patterns and severity among motorcyclists treated in US emergency departments, 2001–2008: a comparison of younger and older riders. Inj Prev.

[29] Khorshidi A, Ainy E, Soori H (2016). Epidemiological pattern of road traffic injuries among Iranian motorcyclist in 2012. J Saf Promot Inj Prev.

[30] Liu BC, Ivers R, Norton R, Boufous S, Blows S, Lo SK (2008). Helmets for preventing injury in motorcycle riders. Cochrane Database Syst Rev.

[31] Mokhtari AM, Samadi S, Hatami SE, Jalilian H, Khanjani N (2014). Investigating the rate of helmet use and the related factors among motorcyclist in Kerman between 1391–92. J Saf Prom Inj Prev..

[32] Heydari SH, Hoseinzadeh A, Sarikhani Y, Hedjazi A, Zarenezhad M, Moafian G (2013). Time analysis of fatal traffic accidents in Fars Province of Iran. Chin J Traumatol.

[33] Hasanzadeh J, Moradinazar M, Najafi F, Ahmadijouybary T (2014). Trends of mortality of road traffic accidents in Fars Province, southern Iran, 2004–2010 Iranian. Aust J Public Health.

[34] Mohammadian M, Hajare A, Mohammadian HA (2014). Incidence trends of injury and mortality from traffic accidents in urban and suburban areas of Isfahan Province during 2002-2010. J Police Med.

